# Mammary adenectomy followed by immediate reconstruction for treatment of patients with early-infiltrating breast carcinoma: a cohort study

**DOI:** 10.1590/1516-3180.2018.0356220719

**Published:** 2019-10-31

**Authors:** Alfredo Carlos Simões Dornellas de Barros, Heloísa Andrade Carvalho, Felipe Eduardo Martins Andrade, Cristiane da Costa Bandeira Abrahão Nimir, Marcelo Moura Costa Sampaio, Fabiana Baroni Makdissi, Max Senna Mano

**Affiliations:** I MD, PhD. Breast Surgeon, Hospital Sírio-Libanês, São Paulo (SP), Brazil.; II MD, PhD. Radiation Oncologist, Hospital Sírio-Libanês, São Paulo (SP), Brazil.; III MD. Breast Surgeon, Hospital Sírio-Libanês, São Paulo (SP), Brazil.; IV MD. Pathologist, Laboratório Diagnostika, São Paulo (SP), Brazil.; V MD, MSc. Plastic Surgeon, Hospital Sírio-Libanês, São Paulo (SP), Brazil.; VI MD, PhD. Breast Surgeon, Hospital Sírio-Libanês, São Paulo (SP), Brazil.; VII MD, PhD. Oncologist, Hospital Sírio-Libanês, São Paulo (SP), Brazil.

**Keywords:** Breast neoplasms, Mastectomy, Prognosis, Esthetics

## Abstract

**BACKGROUND:**

Use of mammary adenectomy for breast carcinoma treatment remains controversial.

**OBJECTIVE:**

This study aimed to verify the oncological safety of mammary adenectomy and immediate breast reconstruction for treating selected patients with infiltrating breast carcinoma and to evaluate patients’ satisfaction with the reconstructed breasts.

**DESIGN AND SETTING:**

Cohort study conducted among patients treated at Hospital Sírio-Libanês, São Paulo, Brazil.

**METHODS:**

This study was based on 152 selected patients (161 operated breasts) with infiltrating breast carcinoma who underwent mammary adenectomy and immediate breast reconstruction. In all patients, the diameter of the largest focus of the tumor was less than 3.0 cm, the imaging tumor-nipple distance was greater than 2.0 cm and the pathological assessment showed clear margins. The cumulative incidence of local recurrence (LR), recurrence-free survival (RFS) and overall survival (OS) curves were estimated using the Kaplan-Meier method. After at least one year of follow-up, 64 patients were asked about their satisfaction with the reconstructed breast(s).

**RESULTS:**

At a mean follow-up time of 43.5 months, seven cases of LR (4.4%), four distant metastases (2.6%) and five deaths (3.3%) were recorded. The five-year actuarial LR-free survival, RFS and OS were 97.6%, 98.3% and 98.3%, respectively. No cases of nipple-areolar complex recurrence were reported. Forty-one patients (64%) indicated a high level of satisfaction with the reconstructed breasts.

**CONCLUSIONS:**

Mammary adenectomy is a safe and efficacious procedure for selected patients with early-infiltrating breast carcinoma and results in a high rate of patient satisfaction with the reconstructed breasts.

## INTRODUCTION

Mammary adenectomy involves resection of all gross visible glandular tissue, including tissue under the nipple, while preserving the overlying breast skin and the nipple-areolar complex (NAC).^1^ It is an evolving procedure for breast cancer patients who are not considered to be candidates for breast-conserving surgery. In mastectomy, the NAC is removed because, in theory, it can harbor neoplastic cells. However, when no initial tumor is located in the central breast region, the frequency of nipple involvement is generally less than 10%. Moreover, with proper selection of patients for mammary adenectomy, NAC relapse rates may be very low.^2^

In 1980, Gentil et al. innovatively performed mammary adenectomy for breast cancer treatment.^3^ Subsequently, Horiguchi et al., Benediktsson et al. and Gerber et al. compared mammary adenectomy favorably with more radical mastectomy for patients.^4^^,^^5^^,^^6^ More recently, other authors suggested that mammary adenectomy may be valid for selected breast cancer cases.^7^^,^^8^^,^^9^^,^^10^^,^^11^

Preservation of the NAC is very important for women’s satisfaction with their physical appearance.^12^^,^^13^^,^^14^ Although it may be tempting for surgeons to offer mammary adenectomy, NAC-sparing surgery should still be recommended only with caution. Persistent uncertainties remain regarding patients’ eligibility, the surgical approach and oncological safety.^2^^,^^11^^,^^15^

There is a paucity of high-quality studies combining all the essential elements of this procedure, such as a standardized surgical procedure, a strict patient eligibility protocol and reporting on long-term oncological outcomes. Here, we present a series of patients who we treated and followed up over a 10-year period at the Sírio-Libanês Hospital, São Paulo, Brazil.

## OBJECTIVES

Our main objective was to report on the oncological safety of mammary adenectomy and immediate breast reconstruction for treating selected patients with infiltrating breast cancer. A secondary objective was to evaluate the patients’ satisfaction with their reconstructed breasts.

## METHODS

### Design, setting and ethics

In this retrospective cohort study, clinical data from selected patients who fulfilled the institutional eligibility criteria for therapeutic mammary adenectomy and immediate breast reconstruction were collected. All patients were operated at Hospital Sírio-Libanês, in São Paulo, Brazil. The research protocol was approved by the institution’s ethics committee (judgment number 10414227; February 18, 2016).

### Inclusion and exclusion criteria

Patients with infiltrating breast cancer were deemed eligible if they fulfilled all of the following inclusion criteria: largest focus of the tumor with less than 3.0 cm in diameter; tumor-nipple distance greater than 2.0 cm according to physical examination and imaging methods; clinically negative axilla or axilla with movable level I-II lymph nodes (cN0-cN1); negative sentinel node biopsy (SNB); and clear surgical margins in intraoperative and definitive analyses.

Patients were excluded if at least one of the following conditions was presented: male breast cancer, clinical evidence of skin/NAC involvement, occult breast cancer, nipple discharge and more than three centers/foci of neoplasia.

### Surgical technique

Two forms of mammary adenectomy were performed: one totally sparing the skin envelope and the other removing a small paddle of skin over the tumor. The surgeries were performed by experienced breast surgeons from the Philanthropic Service of the Mastology Department of the Hospital Sírio-Libanês.

**Figure 1.**
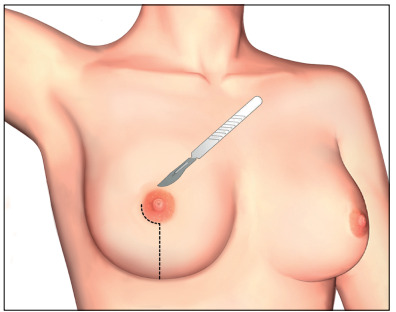
Most frequent incision for mammary adenectomy.

The most frequent form of incision for procedures with total skin maintenance was a vertical radial incision, from the areola to the inframammary fold, going around up to 25% of the areolar circumference (to protect NAC vascularization), in the axillary direction, as shown in Figure 1.

**Figure 2.**
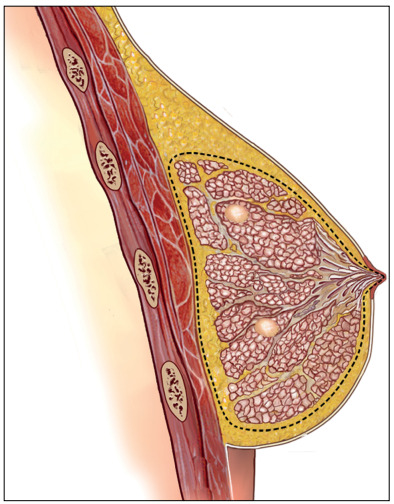
Scheme of the dissection plane for mammary adenectomy in a case with two tumor foci.

**Figure 3.**
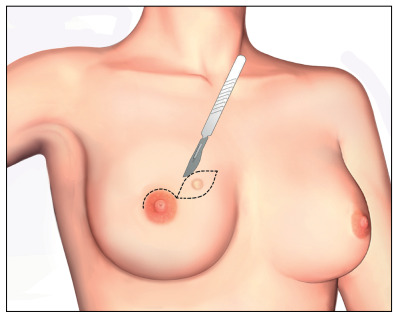
Incision for mammary adenectomy to remove a paddle of skin over the tumor.

**Figure 4.**
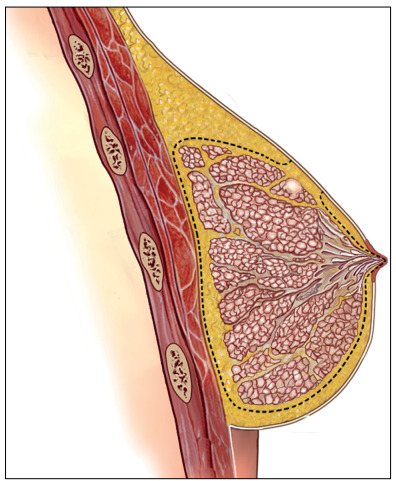
Scheme of the dissection in a case of mammary adenectomy with skin overlying the tumor removed.

The skin flaps were carefully raised using a diathermy knife. It is advisable to make this cut in the thin fascia between the subcutaneous fat and the glandular tissue. The mammary glandular corpus, with the axillary Spence tail, was removed along the pectoralis major muscle fascia. The surgeons had to leave flaps with a thickness of approximately 0.5 cm in the sub-NAC area and 0.5 to 1.0 cm in other parts of the breast (Figure 2). When there was superficial and peripheral neoplasia, located 2.0 cm or more from the areolar border and close to the skin (≤ 2.0 cm in depth), an elliptical skin paddle incision was made in the overlying tumor area. This incision might be extended to the areolar border (Figures 3 and 4).

The perforator branches deriving from the 2^nd^ and 3^rd^ internal thoracic vessels had to be preserved to maintain NAC irrigation. After gland removal, the nipple was inverted, and the ducts arranged inside in the central bundle were excised using a pointed-end knife.

### Surgical margins and nipple duct assessments

During the surgery, the sub-NAC margin was microscopically analyzed by means of imprint cytology and frozen sections (4-µm slices, cut at intervals of 200 µm). If the margin was negative, and remained so according to paraffinized sections, the NAC was preserved. However, if the margin was positive in any of the examinations, the NAC was removed. Nipple ducts were examined only in the definitive analysis. Positive findings indicated that NAC removal was needed, in a second-step procedure.

### Breast reconstruction

Permanent submuscular silicone implants (single-stage procedure) were the mainstay for breast reconstruction. Expander implants and myocutaneous flaps were occasionally used, depending on individual conditions.

Simultaneous mastopexy was performed in women with ptosis, in whom NAC was migrated and centralized in the breast mound.

### Imaging control of the residual tissue and complementary radiotherapy

The remaining fat layer under the skin was evaluated by means of ultrasound or magnetic resonance imaging (MRI), three to six months after the surgery. Conventional fractioned radiotherapy (RT) for the breast/chest wall was delivered when excessive remaining tissue was detected. Supraclavicular lymph nodes were also irradiated, with or without the internal mammary chain nodes, depending on the number of lymph nodes (LNs) affected.

### Adjuvant systemic therapy

Adjuvant chemotherapy and/or hormonotherapy were administered in accordance with contemporary guidelines. All patients with overexpression of human epidermal growth factor receptor-2 (HER-2) received trastuzumab.

### Self-evaluation of esthetic result

At least one year after the surgery, during a consultation, some patients were asked to give their self-evaluation of their reconstructed breast(s). According to their own impression of the final breast silhouette and consistency, the women classified their degree of satisfaction as highly satisfied, satisfied, indifferent, dissatisfied or highly dissatisfied.

### Data acquisition and statistical analysis

Follow-up visits were scheduled for every three months during the first year post-surgery, every six months until the fifth year and annually thereafter. A physical examination was performed during every visit, and breast ultrasonography was performed once a year.

The following oncological outcomes were determined, taking the date of the surgery as the starting date: local recurrence (LR), recurrence-free survival (RFS) and overall survival (OS). Patients alive at the time of the final analysis of the study were censored at the date of their last visit.

Descriptive and frequency analyses were performed. The cumulative incidences of LR, RFS and OS were calculated using the Kaplan-Meier method. The SPSS package version 20.0 (Chicago^11^) was used for statistical analysis.

## RESULTS

Between June 2005 and September 2015, 156 patients underwent mammary adenectomy, comprising 166 breast surgeries (10 patients with bilateral tumors). Four patients (one with bilateral tumors) were lost during the follow-up and, thus, the final numbers considered for the analysis became 152 patients and 161 breasts. LR was estimated according to the number of operated breasts, whereas OS and RFS were estimated according to the number of patients.

The patients’ mean age was 50.2 years (range, 27-84 years). All the patients had infiltrating carcinomas: 81.4% had carcinomas not otherwise specified (NOS); 12.4% had lobular carcinoma; and 6.2% had other subtypes. 41.5% of the population were in clinical stage I, and 58.5% were in stage II. The distribution among pathological stages was as follows: I - 49.6%; II - 39.4%; and III - 11.0%. Most of the patients did not have any lymph node (LN) involvement (66.5%), whereas 24.8% had one to three positive LNs and 8.7% had at least four.

Total skin preservation was performed in 124 breasts (77.0%) and mammary adenectomy with overlying tumor skin removal was performed in 37 breasts (22.9%). Several types of immediate breast reconstruction were used, with predominance of definitive silicone implant placement (136 cases; 84.9%).

**Figure 5.**
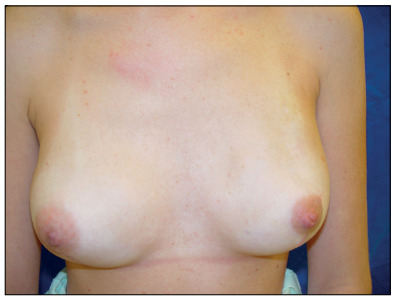
Case in which the patient was highly satisfied with the esthetic result 24 months after the surgery.

**Figure 6.**
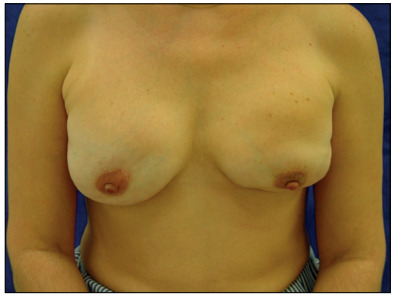
Case in which the patient was highly dissatisfied with the esthetic result 13 months after the surgery.

Sixty-four women were asked about their satisfaction with the reconstructed breast(s). Seventeen (26.5%) were highly satisfied (Figure 5), 24 (37.5%) were satisfied, one (1.5%) was indifferent, 10 (15.6%) were dissatisfied and 12 (18.7%) were highly dissatisfied (Figure 6).

Overall, 56 breasts (34.8%) of 55 patients (one bilateral case) were irradiated. As systemic adjuvant treatment, 70 patients (46.0%) received both chemotherapy and endocrine therapy: 44 (28.9%) received endocrine therapy only; 36 (23.6%) received chemotherapy only; and two (1.3%) received no adjuvant treatment. Trastuzumab was given in combination with chemotherapy in 21 cases (13.8%).

### Oncological outcomes

The length of follow-up was calculated from the date of the surgery until the last follow-up visit or death, whichever came first. The mean length of follow-up was 43.5 months (range, 6-126 months).

At the last data censoring, five deaths (3.3%) had been recorded, among which four (2.6%) related to breast cancer, and one (0.7%) to a non-cancer-related cause. The five-year actuarial estimate of OS was 98.3%.

**Figure 7.**
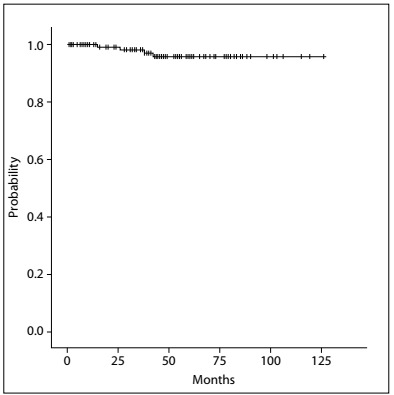
Kaplan-Meier estimates of recurrence-free survival.

**Figure 8.**
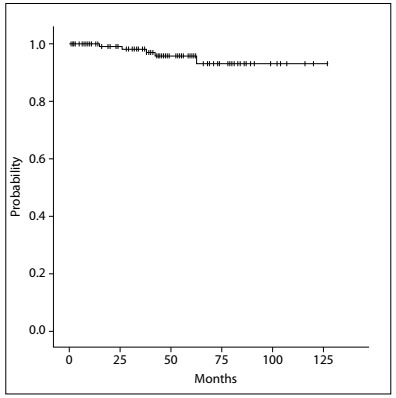
Kaplan-Meier estimates of overall survival.

The crude incidences of first unfavorable events were as follows: 4.4% (seven breasts in seven patients) with LR (one patient presented axillary relapse with associated LR); and 2.6% (four patients) with distant metastasis, among which one also had regional recurrence. There were no cases of NAC recurrence. The five-year actuarial estimate of RFS was 98.3%. The Kaplan-Meier estimates of OS and RFS are shown in Figures 7 and 8.

**Figure 9.**
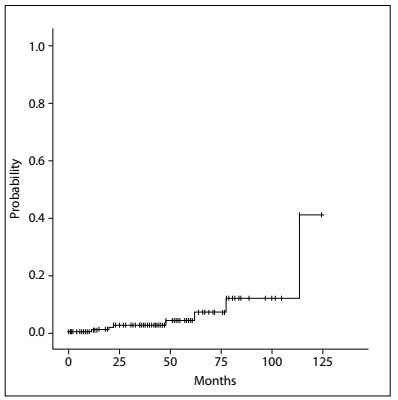
Incidence of local failure events.

LRs, recorded according to the number of breasts operated, were observed for up to 115 months of follow-up. The five-year actuarial estimate of LR-free survival was 97.6% (Figure 9).

We performed a subgroup analysis on 101 patients who had at least three years’ follow-up, in which the cumulative incidence of LR was 5.8% (six cases).

## DISCUSSION

There are concerns about the safety of skin and NAC preservation in patients treated by means of nipple-sparing mastectomies. These concerns relate mainly to the possibility of impairment of local control, as a result of inadequate surgery that leaves behind residual cancer cells. Nevertheless, several studies with high heterogeneity of patient selection and surgical techniques, and with patients presenting indications for RT, have suggested that MA promotes acceptable oncological results.^16^^,^^17^^,^^18^^,^^19^

Our results add to the available literature in terms of reassuring the scientific community about the favorable outcomes among selected BC patients undergoing MA. We observed an LR rate of 4.4%, with no cases of NAC recurrence. In addition, low rates of distant recurrence and breast cancer-related deaths were reported in our cohort (2.6% for each). It is likely that the favorable oncological results obtained in this study can be attributed to proper preoperative patient selection, meticulous sub-NAC margin assessments and adequate operative technique, performed by a homogeneous group of senior breast surgeons at a major cancer reference center.

A paradigm shift from radical surgery toward personalized procedures has evolved over the last few decades. Mammary adenectomy should be a valid alternative for women who are opting for “maximal surgery” instead of breast-conserving surgeries, especially in cases associated with one or more of the following conditions: hereditary breast cancer, young age (≤ 35), tumor multifocality/multicentricity, suspicious diffuse microcalcifications, large tumor in a very small breast, difficulty in achieving intraoperative clear margins in segmental resections or contraindication for RT.

The indication for bilateral mammary adenectomy is sometimes considered. Modern genetic sequencing that allows identification of mutations in suppressor genes of carcinogenesis has strengthened its indication for primary tumor management with concurrent contralateral prophylactic surgery, especially in young patients or in women with a family history of breast cancer. The advantages of the dual procedure derive from its psychological and quality-of-life benefits and maintenance of breast symmetry.^20^ However, this group of patients at high genetic risk of bilateral disease accounts for only a small proportion of the cases. For the average patient, the perception of the risk of contralateral breast cancer is often overestimated. We believe that extensive discussion after patients have been given comprehensive information and reassurance by a multidisciplinary team is a mandatory step before any decision is made.

One essential prerequisite for sparing the NAC is a safe tumor-nipple distance. Our inclusion criteria for mammary adenectomy entailed a distance greater than 2.0 cm, measured by physical examination and imaging methods. This can aid the surgeon in selecting suitable cases for mammary adenectomy. The tumor-nipple distance measured using MRI and the intraoperative pathological margin assessment are the most accurate predictors of occult NAC involvement.^21^^,^^22^^,^^23^^,^^24^

The small ducts inside the nipple are arranged in a central bundle. This configuration enables nipple duct excision, which is deemed advisable.^25^ When positive nipple ducts are found, in either the intraoperative or the postoperative report, NAC excision is required.

The most important step in the surgical approach relates to the skin flap thickness, which should be less than 5.0 mm directly under the areola and should gradually increase from 5.0 to 10.0 mm toward the gland periphery. A delicate surgical plane is usually achievable at the level of the superficial fascial layer between the subcutaneous fatty tissue and breast parenchyma.^26^ Torresan et al. reported that the remnant terminal ductal-lobular units were significantly associated with skin flaps thicker than 5 mm (81.3% versus 46.2%), in specimens from skin-sparing mastectomies.^27^ Since the cosmetic result are improved through preservation of a large subcutaneous tissue pad beneath the skin, one challenge faced by the surgeon is to achieve a balance between radicality and esthetics.

The role of RT among patients treated by MA is a matter of controversy. On the one hand, radiation protects the NAC and adjacent tissue against recurrence, but on the other hand, it may cause dermatitis, contour asymmetry, capsular contracture and implant extrusion.^7^^,^^28^ Most likely, the majority of the patients with early-stage cancers treated by means of mammary adenectomy do not need adjuvant irradiation, except perhaps when postoperative imaging shows an excess of remaining tissue or in cases with more than three LNs affected.

Concerning the women’s satisfaction with their breasts, most of our patients (64%) who responded to the satisfaction survey felt highly satisfied or satisfied. This may be considered to be a fair result, but it highlights the importance of discussing the variable of cosmetic outcomes before making the decision to opt for this surgery. Among 100 patients (117 procedures), Corso et al. found satisfaction with the breasts in 79 cases and satisfaction with the nipples in 31.^29^ High expectations regarding the final cosmetic result should be discouraged.

Moreover, some complications may occur. The complications of mammary adenectomy with immediate reconstruction include flap and/or NAC necrosis, epidermolysis, implant loss, asymmetry, capsule contracture, infection and wound dehiscence. The factors that predispose towards complications are smoking, comorbidities, ptotic breast and periareolar incision.^29^ In a systematic review of the literature with a pooled analysis on 12,358 procedures, Headon et al.^30^ found a nipple necrosis rate of 5.9% and an overall complication rate of 22.3%. In that study, they observed that the rates of complications decreased over time, and this was attributed to improving surgeon expertise. Cutaneous hypoesthesia was very common. All these possibilities need to have been previously addressed.

Our study was not without limitations. There was no control group and the study was conducted on a relatively small number of patients. Nonetheless, despite such limitations, we were able to provide additional insights into personalized surgical treatment of breast cancer.

## CONCLUSIONS

From the current analyses, we were able to conclude that mammary adenectomy preserving the NAC with a minimal amount of remaining glandular tissue, followed by immediate breast reconstruction, was a safe surgical option for selected patients presenting early-infiltration breast carcinoma. Among our patients, there was a high rate of satisfaction with the reconstructed breast(s).
